# Evolutionary Significance of *Wolbachia*-to-Animal Horizontal Gene Transfer: Female Sex Determination and the *f* Element in the Isopod *Armadillidium vulgare*

**DOI:** 10.3390/genes8070186

**Published:** 2017-07-21

**Authors:** Richard Cordaux, Clément Gilbert

**Affiliations:** Laboratoire Ecologie et Biologie des Interactions, Equipe Ecologie Evolution Symbiose, Université de Poitiers, UMR CNRS 7267, Bât. B8, 5 rue Albert Turpin, TSA 51106, 86073 Poitiers CEDEX 9, France; clement.gilbert@egce.cnrs-gif.fr

**Keywords:** horizontal gene transfer (HGT), terrestrial isopod, *Wolbachia*, bacterial endosymbiont, selfish genetic element, sex ratio distorter, feminization, sex determination, sex chromosome

## Abstract

An increasing number of horizontal gene transfer (HGT) events from bacteria to animals have been reported in the past years, many of which involve *Wolbachia* bacterial endosymbionts and their invertebrate hosts. Most transferred *Wolbachia* genes are neutrally-evolving fossils embedded in host genomes. A remarkable case of *Wolbachia* HGT for which a clear evolutionary significance has been demonstrated is the “*f* element”, a nuclear *Wolbachia* insert involved in female sex determination in the terrestrial isopod *Armadillidium vulgare*. The *f* element represents an instance of bacteria-to-animal HGT that has occurred so recently that it was possible to infer the donor (feminizing *Wolbachia* closely related to the *w*VulC *Wolbachia* strain of *A. vulgare*) and the mechanism of integration (a nearly complete genome inserted by micro-homology-mediated recombination). In this review, we summarize our current knowledge of the *f* element and discuss arising perspectives regarding female sex determination, unstable inheritance, population dynamics and the molecular evolution of the *f* element. Overall, the *f* element unifies three major areas in evolutionary biology: symbiosis, HGT and sex determination. Its characterization highlights the tremendous impact sex ratio distorters can have on the evolution of sex determination mechanisms and sex chromosomes in animals and plants.

## 1. Introduction

It is now well-established knowledge that the horizontal transfer of genetic material (i.e., DNA acquired by an organism in the absence of reproduction) is an important driver of prokaryote evolution [1,2]. The accumulation of genomic information within the past years has revealed that horizontal transfer also occurs in eukaryotes, albeit not as commonly as in prokaryotes [3,4]. In animals, most cases of recent horizontal transfer reported so far involve transposable elements [5,6,7] and sequences derived from mitochondria [8] and bacteria [9,10]. A substantial and increasing quantity of adaptive incidences of horizontal gene transfer (HGT) from bacteria to animals have been reported. Striking examples include, but are not limited to, the acquisition of enzymes enabling phytophagy [11,12] and heme biosynthesis [13] in several nematodes and arthropods, and toxins conferring antibacterial immunity in multiple organisms [14,15]. However, the mechanisms underlying these HGT events are unknown, and it remains unclear how bacterial DNA can be integrated into the sequestered germ cells of animals. In this context, it is noteworthy that many of the incidences of bacteria-to-animal HGT reported so far involve intracellular bacterial endosymbionts [9]. Some of these bacteria are maternally transmitted through oocytes, implying that they reside for at least part of the time in the female germline of their animal hosts. Such a particular ecological relationship provides ample scope for bacteria-to-animal heritable HGT.

Many of the cases of endosymbiont-to-animal HGT reported so far have involved *Wolbachia* bacteria. *Wolbachia* endosymbionts belong to the order of Rickettsiales within alphaproteobacteria. They are intracellular bacteria which infect a wide range of arthropods and nematodes [16,17]. Recent estimates indicate that 40–50% of terrestrial arthropods are infected with *Wolbachia*, suggesting that it is the most prevalent endosymbiont on earth [18,19]. *Wolbachia* endosymbionts are maternally transmitted from one host generation to the next through egg cytoplasm. This mode of inheritance explains the strong tropism of *Wolbachia* for the female germ line of their hosts [20] and offers an increased likelihood for the occurrence of HGT. *Wolbachia*-to-host HGT was initially reported from a beetle [21] and a filarial nematode [22]. Subsequently, a large-scale study encompassing multiple arthropod and nematode genomes has demonstrated that *Wolbachia*-to-host HGT is distributed on a large scale [23]. This conclusion has since been confirmed by the discovery of *Wolbachia*-to-host HGT in many more species (e.g., [24,25,26], reviewed in [9,17]).

What is the evolutionary significance of *Wolbachia* sequences being integrated into host genomes? There are several examples of *Wolbachia-*like transferred genes leading to new functions in the nuclear genomes of an aphid [27], a mealybug [28] and a stink bug [29]; however, in all of these cases, it is unclear whether HGT involved *Wolbachia* per se or a member of the Rickettsiales related to *Wolbachia*. Another example is a salivary gland surface protein shared by *Wolbachia* and mosquitoes [30,31]; however, in this case, it is unclear whether the HGT occurred from *Wolbachia* to mosquito or the reverse [30,31]. Sequence analyses identified cases of *Wolbachia*-transferred genes evolving under purifying selection. While this is suggestive of functional cooption, the actual functional relevance of these genes has not been ascertained in general. In fact, most transferred genes unambiguously assigned to *Wolbachia* show signs of pseudogenisation, suggesting that they are evolving neutrally [23,32,33]. Furthermore, while *Wolbachia* inserts are sometimes expressed according to life stage-specific [34] or tissue-specific [35] patterns, they are in most cases transcribed at very low levels, if at all [23,32,33]. Interpreting transcription patterns may be tricky because the absence of transcription under specific conditions does not necessarily mean that the transferred gene is permanently inactive with respect to transcription. In addition, the evidence for transcription does not necessarily indicate biological function, because no criteria exist for the definition of biologically relevant levels of transcription. Moreover, RNA and protein expression levels are not correlated.

**Figure 1:**
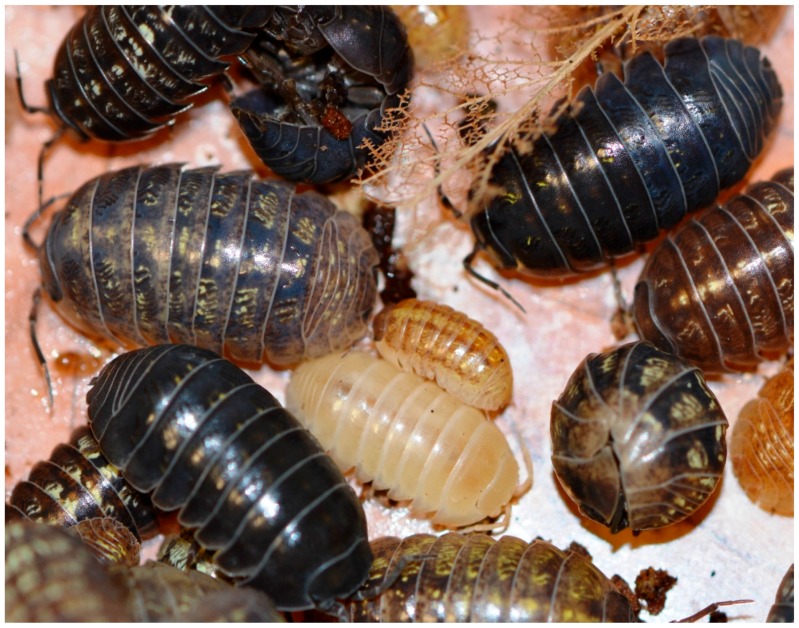
Photography of the common pill-bug *Armadillidium vulgare* (Crustacea, Isopoda, Oniscidea). Males and females are shown. Although quite variable, males tend to be darker than females (the white individual being an albino). Image courtesy of Isabelle Giraud.

The direct functional characterization of *Wolbachia*-transferred genes is not a trivial task. One way would be to clone, express and biochemically characterize proteins of interest. However, prior to the accumulation of deleterious mutations, a transferred gene could apparently retain its original function for some time without having a relevance in its new biological context [9]. Alternatively, it has been proposed that “the gold standard for determining functionality for genes resulting from HGT is a phenotype that correlates to the presence of those genes” [9]. A remarkable case of *Wolbachia* HGT meeting the gold standard proposed by Dunning Hotopp [9] has recently been characterized. This nuclear *Wolbachia* insert, termed the “*f* element”, is involved in female sex determination in the terrestrial isopod crustacean *Armadillidium vulgare* [36] (Figure 1). In this review, we first provide a background for the *A. vulgare*/*Wolbachia* model; then, we offer an account of the current state of knowledge on the *f* element; and, finally, we identify outstanding questions to further our understanding of this topic.

## 2. Feminizing *Wolbachia* and Sex Determination in *Armadillidium vulgare*

In arthropods, *Wolbachia* endosymbionts often manipulate host reproduction to enhance their own vertical transmission. This is achieved by way of four different mechanisms: cytoplasmic incompatibility, parthenogenesis induction, male killing and the feminization of genetic males [16,37]. Of particular relevance here is the feminization of genetic males, which consists of a sex ratio distortion of host progenies in favor of females through the conversion of genetic males into phenotypic females [37,38,39,40]. Feminization has been reported in some insects [41,42,43,44] but is most widespread in terrestrial isopod crustaceans, including the common pill-bug *A. vulgare* [45,46,47].

**Figure 2:**
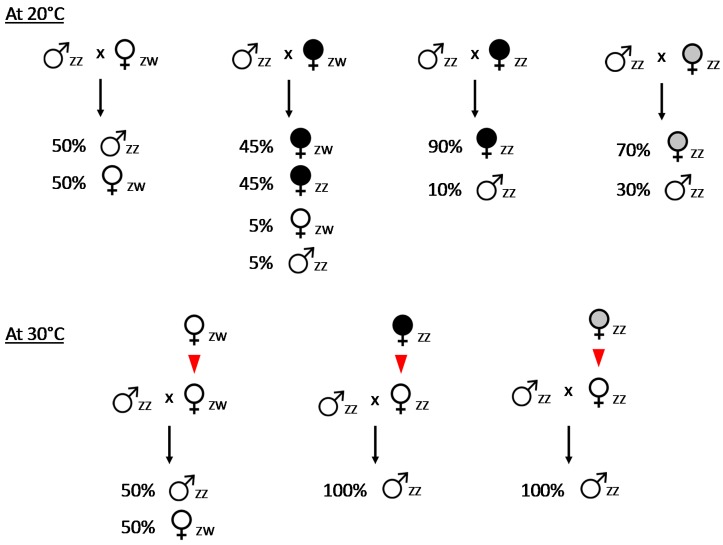
Crosses involving *Armadillidium vulgare* individuals carrying *Wolbachia* (black), the *f* element (grey) or none (white), at 20 °C (top) and 30 °C (bottom). ZZ/ZW: the homo/heterogametic status of individuals. Red triangles denote the elimination of the thermosensitive *Wolbachia* and the *f* element from mothers at 30 °C. *Wolbachia* and *f* element transmission rates from mother to offspring at 20 °C are assumed to be 90% and 70%, respectively. *Wolbachia* and *f* element elimination rates from mothers at 30 °C are assumed to be 100%, although some variation may occur depending on experimental conditions.

In *A. vulgare*, genetic sex determination follows female heterogamety: females have ZW sex chromosomes and males have ZZ sex chromosomes [48]. Crosses between genetic males and females produce progenies with balanced sex ratios, in accordance with the Mendelian transmission of sex chromosomes (Figure 2). However, some females regularly produce highly female-biased progenies (80–90%) and this trait is maternally inherited. Importantly, this phenomenon occurs without differential mortality between sexes, indicating that male-biased mortality can be discarded as an explanation. Instead, it has been shown that this phenotype is caused by the feminizing *Wolbachia* endosymbionts [45]. Zygotes inheriting feminizing *Wolbachia* endosymbionts develop a female phenotype, whatever their genotype [37,38,39,40]. In particular, ZZ genetic males carrying *Wolbachia* are converted into phenotypic females, which in turn produce female-biased progenies (Figure 2). As *Wolbachia* bacteria are thermosensitive, raising infected females at 30 °C eliminates *Wolbachia* [49]. When such heat-treated females are crossed with ZZ genetic males, they produce highly male-biased progenies [50,51] (Figure 2). By contrast, temperature has no effect on the sex ratio of progenies of ZW genetic females [50]. This experiment thus provides direct evidence that infected females are indeed ZZ genetic males feminized by *Wolbachia*.

The molecular mechanism underlying feminization is unknown. However, the androgenic gland (producing the androgenic hormone responsible for male development) generally does not differentiate during the development of ZZ genetic male embryos infected by *Wolbachia* and, as a result, individuals develop as females [52,53]. However, partial feminization sometimes occurs, leading to infected individuals developing as intersex adults. Intersex phenotypes range from fertile females with tiny male secondary sexual characters to sterile males with female genital apertures [54]. These individuals start developing as males (according to ZZ sex chromosomes) before their development shifts at some point to female development (presumably following the action of *Wolbachia*). It has been proposed that incomplete feminization may be linked to an insufficient *Wolbachia* density [55].

An important evolutionary consequence of the presence of feminizing *Wolbachia* in *A. vulgare* is the elimination of the W sex chromosome in infected populations. Indeed, while ZW genetic females produce daughters 50% of the time (ZW), the progenies of *Wolbachia*-infected ZZ females are typically composed of daughters at a rate of 80–90% (ZZ) (Figure 2). Assuming the equal fecundity of both female types, theoretical models predict the extinction of ZW females at equilibrium [56,57] and field observations have confirmed this prediction [58,59]. It follows that sex determination is under the control of *Wolbachia* in infected populations. Indeed, all individuals are ZZ genetic males; those inheriting *Wolbachia* develop as females and *Wolbachia*-free individuals develop as males. As the *Wolbachia* transmission rate is high and stable across generations (80–90%), the progenies of infected females are highly biased towards females. Effectively, this means that the female sex-determining factor shifts from nuclear (W sex chromosome) to cytoplasmic (*Wolbachia*) localization. Thus, the *A. vulgare*/*Wolbachia* system is a perfect example of cytoplasmic sex determination [37,38,39,40].

## 3. Characterization of the *f* Element of *Armadillidium vulgare*

In addition to ZW genetic females (producing progenies with balanced sex ratios) and ZZ phenotypic females carrying feminizing *Wolbachia* (producing female-biased progenies), a third category of females has been found in *A. vulgare*: females producing female-biased progenies in the absence of *Wolbachia* infection [60]. Male-biased progenies obtained from heat-treated females and the crossings of sex-reversed females provided evidence that these females are ZZ genetic males converted into females by a thermosensitive feminizing agent (Figure 2), which was termed the “*f* element” [61]. There are two major features distinguishing females carrying the *f* element from those carrying *Wolbachia*: the stability of the sex ratio bias in progenies and the mode of inheritance of the feminizing factor (Table 1).

**Figure 3:**
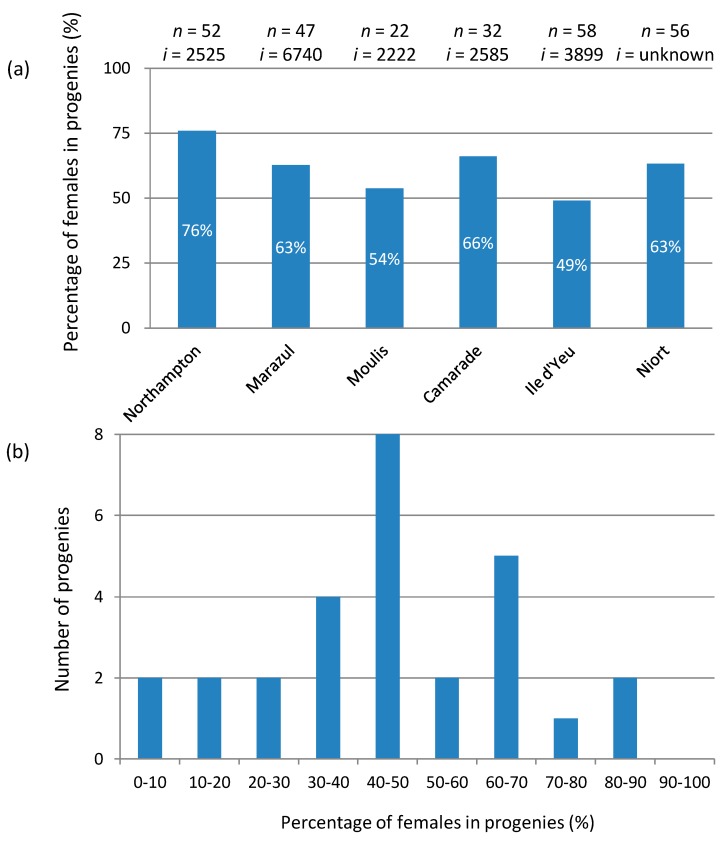
Variations in female percentage in progenies of *Armadillidium vulgare* females carrying the *f* element at different population levels. (**a**) Variation among six natural populations (data from [59,62]); *n* is the number of progenies, while *i* is the total number of individuals. The locations of the populations were the United Kingdom (Northampton), the Canary Islands in Spain (Marazul), and France (Moulis, Camarade, Ile d’Yeu and Niort). The average female percentage across the six populations is 62%. (**b**) Variation within a single population. Distribution of 28 progenies from females sampled in Niort, France (data from [62]), according to female percentage.

**Figure 4:**
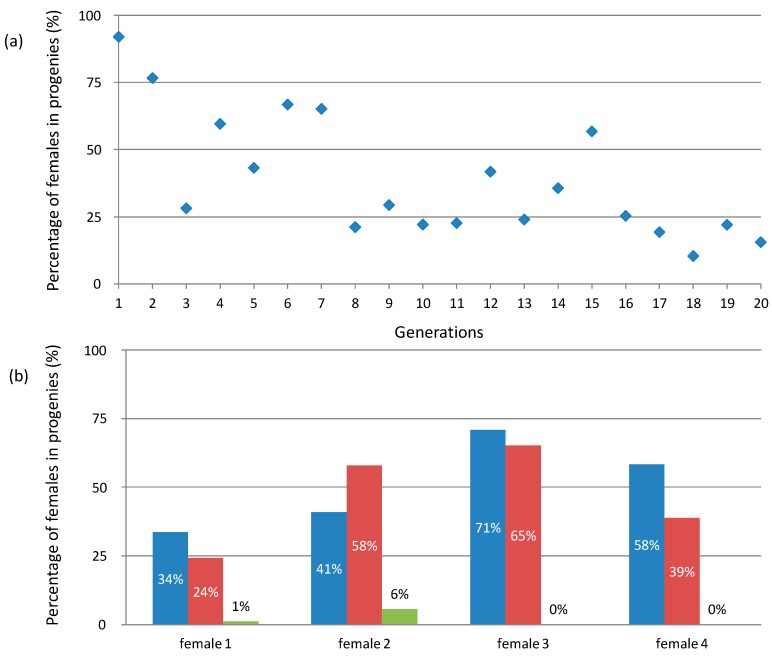
Variation in female percentage in progenies of *Armadillidium vulgare* females carrying the *f* element at the genealogical level. (**a**) Variation in an isofemale line across 20 generations (unpublished results from our laboratory line WXf from 1990 to 2010). (**b**) Variation in three successive progenies of four females (1–4) mated with single males (data from [58]). The first, second and third progenies of each female are shown in blue, red and green, respectively.

A sharp contrast between the *f* element and *Wolbachia* is that, while sex ratio bias induced by *Wolbachia* is very stable across generations (consistently within the range of 80–90% females), sex ratio bias induced by the *f* element is very unstable. While it is within the range of 60–70% females on average [59,62], there is considerable variation between females among (Figure 3a) and within (Figure 3b) populations. At another scale, monitoring iso-female lines has also revealed substantial differences in the sex ratios of progenies between generations (Figure 4a). Even more strikingly, the proportion of females often decreases in the successive progenies of mothers mated with single males (females can store sperm and produce new progenies without multiple matings) (Figure 4b), to the extent that the sex ratio can sometimes vary from 100% to 0% females in the course of three successive progenies [61]. These characteristics suggest an apparent non-Mendelian transmission of the *f* element and imply that the original sex chromosomes are expressed normally in embryos that do not inherit the *f* element [61]. If correct, this interpretation provides additional support for the notion that *f*-carrying females are ZZ genetic males feminized by the *f* element.

The *f* element and *Wolbachia* also differ in their mode of inheritance (Table 1). While *Wolbachia* transmission is exclusively maternal, the *f* element is mainly (albeit not exclusively) inherited maternally; the *f* element can occasionally be transmitted by males [61,63]. The inheritance of the *f* element through males is highly unstable and apparently follows a non-Mendelian pattern [61]. Paternal inheritance of the *f* element can be explained by the existence of a masculinizing gene, termed *M* [64]. It has been shown that the *M* gene is epistatic to the *f* element, i.e., it overrides the feminizing effect of the *f* element and restores a male phenotype [65]. The *M* gene is a dominant autosomal locus, which is also epistatic to the W sex chromosome [64]. In contrast, the *M* gene is not epistatic to *Wolbachia* (Table 1) [65]. While intersex individuals are rare in lines only carrying the *f* element [63,66], the co-segregation of the *M* gene and the *f* element sometimes leads to intersex individuals, termed ♂og, which are fertile males possessing rudimentary female genital apertures [64,66]. These individuals start differentiating as females (presumably following the action of the *f* element) and then shift to male differentiation (presumably following the action of the *M* gene) [64]. Such ♂og individuals are also occasionally produced under the co-segregation of the *M* gene and the W sex chromosome, indicating that the ♂og intersex phenotype is a signature of the *M* gene and not of the *f* element or the W sex chromosome [64,65].

As for *Wolbachia*, the molecular mechanism underlying feminization induced by the *f* element is unknown. Interestingly, the *f* element and *Wolbachia* differ in their ability to induce intersex phenotypes: they occur rarely for the *f* element (in the absence of the *M* gene) but are not uncommon for *Wolbachia* [54,66] (Table 1). Moreover, young *f*-carrying females can be experimentally reversed into males through the implantation of male androgenic glands [61]; by contrast, it is impossible to reverse young females infected by *Wolbachia* into males using this experimental procedure [66]. These observations suggest that the *f* element and *Wolbachia* may not necessarily have the same molecular targets to induce or maintain, or both, host feminization.

A prerequisite to understanding the molecular mechanism of *f*-induced feminization is to characterize the molecular nature of the *f* element. Genetic males inoculated with tissues carrying the *f* element are not feminized, whereas they are feminized when inoculated with tissues carrying cytoplasmic *Wolbachia* [61]. This is consistent with electron microscopy screenings, which ruled out the presence of cytoplasmic factors (including *Wolbachia*) in the cells of *f*-carrying females [36,38,61]. Instead, together with occasional paternal inheritance, these lines of evidence suggested that the *f* element has a nuclear localization. In 1984, Legrand and Juchault [61] proposed that the *f* element may be a sequence originating from *Wolbachia* which carries feminization information and is located in the nucleus. Evidence supporting a *Wolbachia* origin of the *f* element came from crossing experiments; while studying the descent of a genetic female inoculated with *Wolbachia*, Legrand and Juchault [61] observed the appearance of an *f*-like pattern of transmission after *Wolbachia* failed to be transmitted by a female to its progeny. Legrand and Juchault [61] more specifically hypothesized that the *f* element may be (i) a bacteriophage that would have shifted hosts from *Wolbachia* to *A. vulgare*; or (ii) a *Wolbachia* plasmid or chromosome-piece integrated in the *A. vulgare* genome. However, no bacteriophage or viral particle has ever been observed by electron microscopy in *f*-carrying females, despite extensive investigations [36,38,61]. While these observations suggested that the *f* element is unlikely to be a *Wolbachia* bacteriophage, the possibility remained that the *f* element may be a plasmid or a piece of the *Wolbachia* genome transferred into a host chromosome [67].

To test these hypotheses, the genome of an *f*-carrying female was recently sequenced using the illumina technology [36]. A search for *Wolbachia*-like sequences identified a ~3 Mb insert of a *Wolbachia* genome which was nearly identical at the nucleotide level to the feminizing *Wolbachia* strain *w*VulC of *A. vulgare*. The nuclear localization of the insert was confirmed by its gene content, which is incompatible with cytoplasmic *Wolbachia* cell viability, and by the identification of a junction between the *Wolbachia* sequence and the *A. vulgare* genome. Remarkably, PCR tests and analyses of sequencing depth indicated that the *Wolbachia* insert is fully linked to the female sex and hemizygous, as may be expected for the sex-determining region of the genome [68]. Genetic crosses demonstrated that the *Wolbachia* insert occurred in a ZZ genetic male background, thus ruling out that linkage to female sex and hemizygosity could merely be a by-product of integration in the female sex-determining region of the native W sex chromosome. Instead, the *Wolbachia* insert appears to act as the female sex-determining region of the genome. This is fully consistent with earlier findings indicating that *f*-carrying females are ZZ genetic males converted into phenotypic females by the feminizing *f* element [61]. Taken together, these results demonstrate that the *f* element is a large piece of a feminizing *Wolbachia* genome that has been horizontally transferred in the *A. vulgare* genome [36].

It is remarkable that the original hypothesis of the *f* element as a *Wolbachia*-to-animal HGT was put forward more than 30 years ago [61]; nearly 20 years before the first example of *Wolbachia*-to-animal HGT was reported [21]. In retrospect, this was a quite provocative and visionary proposal, especially considering that, in the early 1980s, a single case of bacteria-to-eukaryote HGT had been described: the transfer DNA (T-DNA) sequence of *Agrobacterium tumefasciens*, the causative agent of crown gall disease in plants [69].

## 4. Outstanding Questions Regarding the *f* Element

The *f* element of *A. vulgare* is a remarkable case of bacteria-to-animal HGT as it has occurred so recently that it was possible to infer the donor of the transfer (feminizing *Wolbachia,* closely related to *w*VulC), the mechanism of integration (a nearly complete genome inserted by micro-homology-mediated recombination) and the evolutionary significance of the transferred sequence (its recruitment as a sex-determining region) [36]. The characterization of the molecular nature of the *f* element offers completely new perspectives on questions related to its involvement in female sex determination, unstable inheritance, population dynamics and molecular evolution.

### 4.1. What Is the Molecular Genetic Basis of Feminization Induced by the f Element?

While the molecular nature of the *f* element has been elucidated, the underlying genetic basis of feminization remains an open question. Given the known feminizing effect of the *Wolbachia* cytoplasmic ancestor of the *f* element, the most intuitive hypothesis is that one or several genes encoded by the *f* element cause feminization. The difficulty in identifying feminization genes lies in the fact that the *f* element is a very large sequence (~3 Mb) containing more than 3000 genes [36]. Unlike most *Wolbachia*-transferred genes previously reported in invertebrates, which show signs of pseudogenisation [23,32,33], most genes are devoid of non-sense mutation and thus potentially functional in the *f* element, owing to the recent occurrence of the HGT. One strategy to help narrow down the list of feminization candidate genes may be to investigate the expression patterns of the genes located in the *f* element. In this regard, it is important to consider that these bacterial genes are now located in a eukaryotic cell context and that the regulation of gene expression varies between prokaryotes and eukaryotes. One may thus expect that very few genes in the *f* element are expressed in the *A. vulgare* nucleus, as previously reported for other *Wolbachia*-transferred genes [23,32,33]. In turn, genes in the *f* element transcribed during *A. vulgare* female sex differentiation stages would constitute prime candidates for an involvement in feminization. Obtaining a short list of candidate genes would then open up the possibility of performing functional tests, which at present cannot be run systematically, on the more than 3000 genes of the *f* element. Such functional tests could include interfering RNAs or CRISPR/Cas9 editing, both of which have been shown to be effective in crustaceans [70,71,72].

While the presence of feminization genes in the *f* element is very likely to explain its feminizing ability, we cannot at present formally discard a mutagenic effect of the *f* element. If this were the case, as *f* element integration occurred in a male genetic background [36], it could disrupt a gene involved in the male sex determination or differentiation cascade, either by direct inactivation or by altering its regulation, thereby inducing the evolution of a switch to female sex determination. Such a positional effect is a plausible explanation in principle, as previously reported in melon, in which the presence or absence of a transposable element insertion controls sex determination [73]. An inspection of the *A. vulgare* genomic sequence flanking the *f* element indicated that integration occurred in a (CCTAA)_n_ microsatellite, ~10 kb away from an endogenous viral element of the *Nimaviridae* family [36]. The next coding DNA sequence is located ~35 kb away from the *f* element and encodes a protein of unknown function. Thus, the hypothesis of a mutagenic effect of the *f* element by gene inactivation upon integration appears to be quite unlikely; it should be noted, however, that only a single genomic sequence flanking the *f* element has been characterized so far. Thus, it is conceivable that the *f* element alters the regulation of a gene involved in sex differentiation or determination located in the other genomic flank. Testing this possibility will require the characterization of the second flanking sequence of the *f* element, which may be achieved by taking advantage of the long sequencing reads enabled by PacBio (Pacific Biosciences, Menlo Park, CA, USA) or MinION (Oxford Nanopore Technologies, Oxford, UK) technologies, for example.

One reviewer of this manuscript has suggested that the *f* element may not be involved in female sex determination at all, but instead may merely be integrated near and genetically linked to a female sex determination gene of the *A. vulgare* genome. Such a scenario is unwarranted for the following reasons. Importantly, it has been demonstrated that the *f* element did not integrate in the female sex-determining region of the native W sex chromosome of *A. vulgare* [36]; the suggested scenario thus requires that another hypothetical neo-W sex chromosome would have evolved just before or after the integration of the *f* element. If so, it would require that a purely nuclear female sex-determining region evolved in the course of a feminizing *Wolbachia* infection and cytoplasmic sex determination; on the contrary, sex ratio selection predicts that the evolution of a purely nuclear sex-determining region should be masculinizing, not feminizing, in the context of nucleo-cytoplasmic conflicts existing in the *A. vulgare*/*Wolbachia* system [74,75]. It follows that a mere linkage of the *f* element to a hypothetical female sex-determining region is highly unlikely.

In summary, as discussed above, feminization associated with the *f* element is most likely caused either by feminizing genes it carries or the disruption of the male sex determination/differentiation cascade. It is noteworthy that, in both cases, the *f* element is causal in the induction of feminization. Thus, whatever the underlying molecular mechanism of feminization in the *f* element, the genomic locus in which the *f* element is inserted has effectively become a new female sex-determining region and, thus, the *A. vulgare* chromosome that carries the *f* element fulfills the definition of a new W sex chromosome.

### 4.2. How to Explain the Unstable Inheritance of the f Element?

Early studies have highlighted the instability of the *f* element, which was predicted to reflect a combination of a frequent loss from oocytes and recurrent gains from *Wolbachia* bacteria [61]. Under this hypothesis, the apparent non-Mendelian inheritance of the *f* element (i.e., transmission, at a frequency significantly different from 50%, to gametes in hemizygous individuals) would in fact correspond to the Mendelian inheritance (i.e., transmission to 50% of gametes in hemizygous individuals) of multiple independent *f*-like elements coexisting as polymorphic loci in *A. vulgare* populations. This scenario predicts that *f*-like elements should have different genomic locations. Interestingly, the molecular characterization of the *f* element and the flanking genomic region allowed the design of a PCR assay using a marker (named Jtel) spanning the junction between the *f* element and the flanking sequence [36]. This PCR assay enables the testing of the above prediction in natural populations. Preliminary results based on samples from two European countries support the existence of a single *f* element in all populations tested so far (R. Cordaux, unpublished results). Pending confirmation, these results therefore do not support the hypothesis of recurrent losses and gains in explaining the instability of the *f* element.

Another explanation could be that the *f* element follows Mendelian inheritance but its penetrance is unstable, hence producing an apparent unstable pattern of transmission [39]. Incomplete penetrance could explain male-biased progenies, but it cannot account for female-biased progenies; indeed, females hemizygous for the *f* element transmit it to half of their offspring under Mendelian inheritance. Thus, whatever the penetrance of the *f* element, the highest possible proportion of females in progenies is 50% [39]. Furthermore, a pedigree analysis has recently indicated that all individuals carrying the *f* element are females (in the absence of the masculinizing *M* gene), thus providing empirical evidence that penetrance of the *f* element is 100% [36]. In summary, the instability of the *f* element is unlikely to be related to penetrance issues.

The common underlying assumption of the previous hypotheses is that confounding factors have blurred the signal of Mendelian transmission of the *f* element. An alternative explanation is that the *f* element truly exhibits non-Mendelian inheritance; if so, the *f* element could be associated with segregation distortion (also known as meiotic drive), which is a widespread phenomenon in animals [76,77,78]. One possibility is that the *f* element itself possesses the genetic information enabling segregation distortion. Interestingly, recent evidence suggests that *Wolbachia* may cause feminization and segregation distortion in butterflies [79]. Another possibility is that the *f* element does not cause segregation distortion itself but is inserted in an *A. vulgare* genomic region subject to segregation distortion. In this case, the *A. vulgare* locus experiencing distortion was cryptic, because it was not linked to any obvious phenotype. The integration of the *f* element in the genome would have uncovered the distortion effect because of its linkage with feminization, resulting in progenies showing biased sex ratios. The latter explanation may seem more parsimonious than the former, which requires the *f* element to cause two concerted phenotypes (feminization and segregation distortion) which may be quite complex to operate for genes of bacterial origin in eukaryotic cells. More generally, however, segregation distortion implies that driver loci are present in more than half of the offspring of heterozygous individuals [76,77,78]. Thus, while segregation distortion could explain the production of female-biased progenies from females hemizygous for the *f* element, it cannot account for male-biased progenies in which the *f* element is transmitted to fewer than half of the offspring. Should this occur, it would imply that repressors of segregation distortion may also be present in the system. In this context, the apparent evolution of the Mendelian inheritance of the *f* element reported in an *A. vulgare* line from Morocco might indicate that an equilibrium between distortion and repression can be reached [67].

Another type of non-Mendelian genetic element that may be prone to instability in inheritance patterns and may induce sex ratio biases are supernumerary B chromosomes—additional dispensable chromosomes to the standard chromosome complement—which are also widespread in animals [77,80]. Could the *f* element belong to a B chromosome? Cytogenetic analyses of >130 chromosome plates from genetic males and females, females infected with *Wolbachia* or the *f* element, and males carrying the *M* gene from multiple populations all consistently indicated the presence of 27 pairs of chromosomes without any detectable B chromosome [81]. One could argue that the small size of the *f* element (~3 Mb) may have precluded its detection in this study. Ideally, the fluorescent in situ hybridization of metaphasic chromosomes could be used to address this issue. Unfortunately, obtaining chromosome spreads of good quality and in sufficient quantity appears to be very challenging for *A. vulgare* females, and we have not been able to obtain satisfactory results so far despite our efforts (C. Gilbert and R. Cordaux, unpublished results). Another way to assess whether the *f* element may be a B chromosome is to ask whether it possesses molecular properties that are typically expected of B chromosomes, such as copy number variation between cells and an absence of genes [77,80]. Sequencing of the *f* element indicated the presence of >3000 *Wolbachia* genes, and the available flanking sequence also contains multiple predicted coding DNA sequences [36]. Thus, if the *f* element belongs to a B chromosome, then it is an atypical one.

Overall, it appears that the causes of the unstable inheritance of the *f* element remain a mystery for now. The further molecular characterization of the genomic sequences flanking the *f* element on both sides is likely to produce useful information which could help to settle this issue.

### 4.3. What Is the Population Dynamics of the f Element?

Another intriguing question relates to the increase in frequency of the *f* element in the original *A. vulgare* line in which the *f* element arose. Indeed, given the origin of the *f* element, this *A. vulgare* line was necessarily infected with feminizing *Wolbachia*. Theoretical models predict the extinction of the least feminizing factor under a scenario of competition [56]. Nevertheless, despite a lower feminizing effect, the *f* element was able to outcompete *Wolbachia*. A possible explanation for this unexpected outcome is genetic drift, which may have resulted in *Wolbachia* loss by chance, thereby generating a genetic background with the *f* element alone. This is plausible given *Wolbachia* infection dynamics in arthropods, which involves frequent gains and losses [82]. Alternatively, carrying *Wolbachia* may cause a higher fitness cost to hosts relative to the *f* element. Unfortunately, virtually no information is available regarding the fitness cost of the *f* element. By contrast, a deal of evidence supports a fitness cost of *Wolbachia*; indeed, it has been shown that *A. vulgare* females infected by *Wolbachia* exhibit lower reproductive success [83,84], lower immunocompetence [85,86] and lower cognitive capabilities [87] relative to genetic females devoid of *Wolbachia*. Such costs may be sufficient to cause *Wolbachia* loss at individual or family levels, although they may not be high enough to cause *Wolbachia* loss at population level, especially as beneficial effects of *Wolbachia* infection have also been reported in *A. vulgare* [88]. Overall, it is still unclear how the *f* element was able to rise in frequency during its early evolution. Investigations of life history traits associated with individuals carrying the *f* element and comparisons with those carrying *Wolbachia* will shed new light on this issue.

More generally, studies of the distribution of sex-determining factors in natural populations of *A. vulgare* suggest that the most frequent feminizing factor is the *f* element, not *Wolbachia* or the W sex chromosome [58,59,63,89]. Why is the *f* element more frequent than *Wolbachia* in natural populations, despite a lower feminizing effect? As proposed above, this situation may reflect higher fitness costs caused by *Wolbachia* relative to the *f* element at population level. Of relevance is the fact that intersexuality (which may cause sterility) is frequently associated with *Wolbachia* infection, while it is rare with the *f* element (Table 1). This may represent an important shortcoming for *Wolbachia* spread in *A. vulgare* populations relative to the *f* element. The occasional paternal inheritance of the *f* element, which does not apply to *Wolbachia*, is also relevant. This may facilitate the invasion of a new host’s genetic background by the *f* element when compared to *Wolbachia*. A strong limitation to a better understanding of the evolutionary dynamics of the *f* element is that population data are presently too scarce to enable conclusions to be drawn. The recent characterization of the *f* element and the design of diagnostic molecular markers, coupled with the availability of *Wolbachia*-specific markers [36,90], now makes it possible to initiate large-scale studies of the distribution of feminizing factors in *A. vulgare* populations. This will contribute invaluable information to address general questions on the evolution of the *f* element.

### 4.4. What Are the Patterns of Molecular Evolution of the f Element?

A striking result arising from the molecular characterization of the *f* element is that genomic rearrangements mediated by intra-chromosomal recombination appear to be a major signature of the early molecular evolution of this recently evolved sex-determining genomic region [36]. Indeed, the *f* element experienced tens of duplications of variable size (up to 170 kb), which collectively doubled its size since its integration in the *A. vulgare* genome. This is particularly striking considering the paucity of nucleotide substitutions that distinguish the *f* element from cytoplasmic *Wolbachia w*VulC (i.e., only 3.5 substitutions per gene on average). It will be interesting to further dissect the evolution of these genomic rearrangements and the extent of the variability they show in natural populations, because it could provide important information to help understand how the *f* element induces feminization and why the inheritance of the *f* element is so unstable. For example, because of the history of duplications of the *f* element, some genes have been duplicated several times, and perhaps variation in the copy number or the regulation of these genes has consequences on the biology of the *f* element, which could explain variation in its phenotypic expression.

More generally, uncovering the patterns and processes of the molecular evolution of the *f* element may have implications for our understanding of the origin and early evolution of sex chromosomes. The evolution of sex chromosomes is thought to be triggered by the acquisition of a sex-determining gene on a pair of autosomes [75]. With the additional acquisition of sexually antagonistic genes near this sex-determining gene, a non-recombining region can be established, which then physically extends over time. The non-recombining region accumulates deleterious mutations and transposable elements which ultimately lead to massive gene loss and the heteromorphy (i.e., morphological differentiation) of the sex chromosomes. This long-term degeneration of sex chromosomes is well characterized, thanks to the analysis of old sex chromosomes such as the human Y chromosome [91]. Nevertheless, degenerated sex chromosomes have lost the molecular signatures of their incipient evolution, and understanding the early evolution of sex chromosomes requires the analysis of young, recently evolved sex chromosomes, such as the chromosome hosting the *f* element of *A. vulgare*. A key step in the early evolution of sex chromosomes is recombination suppression in the sex-determining region of the genome [68,75]. It has been proposed that structural genomic rearrangements could constitute a major mechanism that initiates recombination arrest [68,75]. The *f* element provides empirical support for this hypothesis, as it was apparently integrated in the *A. vulgare* genome as a single, large (~1.5 Mb) genomic sequence, which, after many internal duplications and rearrangements, inflated into a shuffled ~3 Mb region [36]. Furthermore, the hemizygosity of the *f* element in the *A. vulgare* genome [36] has *de facto* generated a situation in which homologous recombination cannot operate. It is noteworthy that the occasional paternal inheritance of the *f* element may offer opportunities for the production of females homozygous for the *f* element that might recombine. However, the extensive internal genomic rearrangements experienced by the *f* element might affect the possibility of homologous recombination in these homozygous females. In summary, investigations into the nature and variability of the structural rearrangements that govern the molecular evolution of the *f* element are likely to offer new insights into early sex chromosome evolution in general.

## 5. Conclusions and Perspectives

The *f* element of *A. vulgare* represents a remarkable example of *Wolbachia* HGT, for which a clear correlation with a phenotype has been uncovered [36]. Being involved in female sex determination, the *f* element unifies three major areas in evolutionary biology which are currently subjects of intense research: symbiosis, HGT and sex determination. Notably, the identification and study of the *f* element has broad implications for our understanding of the evolution of sex-determining mechanisms and sex chromosomes in animals and plants. Indeed, the *f* element supports an evolutionary scenario in which *Wolbachia* generated a turnover of sex chromosomes in *A. vulgare*, first by causing the loss of the original W sex chromosome under cytoplasmic sex determination, then by introducing a novel sex-determining sequence by way of HGT, resulting in the evolution of a new W sex chromosome [36]. In addition, sex ratio distorters such as *Wolbachia* and the *f* element induce genetic conflicts leading to strong selective pressures, thereby promoting the evolution of nuclear repressors of feminization (i.e., masculinizing genes) that can restore balanced sex ratios [37]. Such genes may evolve as new male sex-determining genes, which would thus establish novel Y sex chromosomes, resulting in another type of turnover of sex chromosomes [92]. In this context, it has been proposed that the masculinizing *M* gene of *A. vulgare* may have evolved to repress the feminizing effect of the *f* element [65]. Thus, *Wolbachia* and other sex ratio distorters can have a tremendous impact on the evolution of host sex determination mechanisms. This impact is unlikely to be restricted to *A. vulgare*, because *Wolbachia* endosymbionts are widespread in terrestrial isopods (i.e., the suborder containing *A. vulgare*) [90,93]. Evidence indicates that multiple turnovers of terrestrial isopods and that sex chromosomes are apparently very young in many species, which is consistent with the role of *Wolbachia* in shaping sex determination systems at the scale of an entire animal group [94,95]. Considering that sex ratio distorters such as *Wolbachia* and the *f* element are highly diverse and widespread in many animals and plants [75], it is likely that they have had an important impact on the evolution of sex determination systems not just in *A. vulgare* and other terrestrial isopods, but more generally in a broad spectrum of sexual organisms.

## Figures and Tables

**Table 1:** Comparison of various biological features of feminizing *Wolbachia* and the *f* element in the terrestrial isopod *Armadillidium vulgare*.

Biological Feature	Feminizing *Wolbachia*	*f* Element
Conversion of genetic males into phenotypic females	Yes	Yes
Presence of cytoplasmic microorganisms	Yes (bacteria)	No
Thermosensitivity	Yes	Yes
Mendelian inheritance	No	No
Mode of inheritance	Exclusively maternal	Mainly maternal, occasionally paternal
Stability of sex ratio bias in progenies	Yes (80–90% females)	No (60–70% females on average, but ranges from 0% to 100% females)
Presence of intersex individuals in progenies	Yes (up to 25%)	Rare (<1%)
Experimental reversal into males by implantation of androgenic gland	Failure	Success
Epistasis with masculinizing *M* gene	*Wolbachia* > *M* gene	*M* gene > *f* element
